# The modified Glasgow prognostic score serves as a robust predictor of unplanned readmission and 1-year mortality in lung cancer patients receiving immune checkpoint inhibitors

**DOI:** 10.3389/fonc.2025.1698848

**Published:** 2026-01-21

**Authors:** Fengwang Xue, Ruoqing Lu, Cailian Wang, Qilian Xiong, Ying Liu, Shengmin Guo, Bo Deng

**Affiliations:** 1 School of Nursing, Southwest Medical University, Luzhou, China; 2 Department of Neurosurgery, the Affiliated Hospital of Southwest Medical University, Luzhou, China; 3 Department of General Surgery, Luzhou People’s Hospital, Luzhou, China; 4 Emergency Department & ICU, Chongqing Hospital of Traditional Chinese Medicine, Chongqing, China; 5 Oncology Department, Chongqing Hospital of Traditional Chinese Medicine, Chongqing, China; 6 Department of Nursing, The Affiliated Hospital of Southwest Medical University, Luzhou, China

**Keywords:** immune checkpoint inhibitors, lung cancer, modified Glasgow prognostic score, prognosis, unplanned readmission

## Abstract

**Background:**

The modified Glasgow Prognostic Score (mGPS), which reflects the degree of systemic inflammation and nutritional status, is associated with prognosis in various common malignancies. However, its association with 30-day unplanned readmission and 1-year mortality in stage III-IV lung cancer (LC) patients remains unvalidated. This study aimed to evaluate the prognostic value of mGPS in stage III-IV LC patients receiving immune checkpoint inhibitors (ICIs).

**Methods:**

In this retrospective study, 209 patients diagnosed with stage III-IV LC who underwent ICI therapy between January 2023 and May 2024 were included. Patients were stratified based on mGPS scores into three risk categories: low-risk (0 points), intermediate-risk (1 point), and high-risk (2 points). Kaplan-Meier analyses, multivariate Cox proportional hazard regression, and subgroup analyses were employed to assess primary outcomes.

**Results:**

Among the enrolled patients, the rates of 30-day unplanned readmission and 1-year mortality were 35.4% (74/209) and 11.0% (23/209), respectively. Kaplan-Meier analysis indicated significantly elevated cumulative incidences of 30-day unplanned readmission and 1-year mortality in the high-risk group relative to intermediate- and low-risk groups (log-rank *p* < 0.001). Adjusted multivariable Cox regression revealed that each 1-point increase in mGPS conferred a 72% higher risk of 30-day unplanned readmission (*HR* 1.72, 95%*CI* 1.25-2.38, *p* = 0.001) and a 117% higher risk of 1-year mortality (*HR* 2.17, 95%*CI* 1.15-4.10, *p* = 0.017). Additionally, compared with low-risk patients, those in the high-risk group experienced a 198% increase in the risk of 30-day unplanned readmission (*HR* 2.98, 95% *CI* 1.56-5.69, *p* = 0.001) and a 366% increase in 1-year mortality risk (*HR* 4.66, 95% *CI* 1.33-16.35, *p* = 0.017). Trend tests confirmed that the risk of adverse outcomes rose steadily with increasing mGPS risk category. Subgroup analyses demonstrated that the prognostic effect of mGPS was consistent across age, TNM stage, metastatic status, and nutritional condition (*p* for interaction > 0.05).

**Conclusion:**

Higher mGPS scores significantly correlate with elevated risks of both 30-day unplanned readmission and 1-year mortality among LC patients receiving ICI therapy. Routine mGPS monitoring may warrants further evaluation in prospective multicenter validation studies to inform prophylactic interventions.

## 1 Introduction

Lung cancer (LC) remains among the malignancies associated with the highest morbidity and mortality globally (1). In 2022, approximately 2.48 million new cases were diagnosed worldwide, and LC accounted for approximately 1.80 million deaths. Notably, nearly 70% of cases are diagnosed at an advanced stage (2). Although ICIs, have markedly improved clinical outcomes for advanced-stage LC patients, only about 20% derive substantial clinical benefit (3). Additionally, frequent unplanned readmissions continue to challenge the management of these patients (4, 5).

Unplanned readmission, defined as any unscheduled hospitalization for the same or a related condition after discharge, increases financial and psychological burdens, elevates mortality risk, and contributes to excessive healthcare resource use (6). In 821 LC patients followed for a median of 32.8 months, Sánchez et al. reported 1,186 unplanned readmissions, generating €6.28 million in direct medical costs (7). Research from Taipei, China, showed that older patients with unplanned readmission had an 11.78−fold higher 1-year mortality risk after discharge (8). Consequently, readmission rates have become a key metric for assessing hospital care quality (9).

Mounting evidence highlights the predictive value of inflammation- and nutrition-related biomarkers concerning prognosis and disease progression in malignant tumors. The modified Glasgow Prognostic Score (mGPS), a practical, cost-effective inflammatory scoring system derived from serum albumin (ALB) and C-reactive protein (CRP), comprehensively reflects systemic inflammatory and nutritional conditions. Previous studies have validated its prognostic utility in rectal cancer (10), ovarian carcinoma (11), soft tissue sarcomas (12), and in non-small cell lung cancer patients treated with ICIs (13). Moreover, mGPS has shown promise in predicting therapeutic response to immunotherapy (13, 14).

Current literature predominantly evaluates the association of mGPS with overall survival and progression-free survival outcomes in LC (15–19), whereas investigations addressing its relationship with short-term unplanned readmissions (within 30 days post-discharge) remain sparse. During this transitional recovery period following hospitalization, patients experience ongoing physiological adaptation and healing, rendering them particularly susceptible to disease exacerbations related to cancer treatments, subsequently increasing readmission risk (20). Therefore, precise identification of risk predictors for early readmission episodes is clinically imperative.

This study explored the association of mGPS with 30-day unplanned readmission and 1-year mortality, aiming to provide an evidence-based approach for the early detection of high-risk patients and facilitate the development of personalized preventive interventions.

## 2 Methods

### 2.1 Study design and patient recruitment

This retrospective cohort analysis included patients diagnosed with stage III-IV LC admitted to the oncology unit of a tertiary medical center in Southwest China from January 2023 through May 2024. Participants met the following eligibility criteria: (1) confirmed malignant LC at stage III or IV via pathology; (2) age of at least 18 years; (3) receipt of ICI monotherapy (e.g., tislelizumab, sintilimab, or toripalimab), without chemotherapy, radiotherapy, targeted therapy, or other anti-cancer interventions in the preceding three months; and (4) availability of complete clinical records.

Exclusion criteria: (1) patients whom the attending physician deemed not medically ready for discharge but who, or whose family members, insisted on leaving against medical advice; (2) readmission within 30 days for planned anti-tumor therapy; (3) receipt of blood transfusion, ALB infusion, or nutritional infusion within the previous 3 months; (4) missing serum CRP or ALB values, required for mGPS calculation.

### 2.2 Data collection

Demographic and clinical variables were obtained from electronic health records. Baseline characteristics comprised age, sex, smoking history, and alcohol use. Disease-specific data included TNM stage, diabetes mellitus, hypertension, coronary artery disease, cerebrovascular disease, distant metastasis, Karnofsky Performance Status (KPS), Nutritional Risk Screening 2002 (NRS 2002) score, and Activities of Daily Living (ADL) score. All biochemical parameters included in this analysis were collected between 06:00 and 08:00 on the morning of discharge, after patients had met discharge criteria following immune-checkpoint inhibitor therapy, and comprised white blood cell (WBC) count, lymphocyte count, neutrophil count, red blood cell count, hemoglobin, platelet count, CRP, ALB, alpha-fetoprotein, carcinoembryonic antigen (CEA), carbohydrate antigen 19-9 (CA 19-9), and carbohydrate antigen 50 (CA50).

### 2.3 mGPS

Consistent with prior work, the mGPS was calculated from serum CRP and ALB levels (21–25). Patients with CRP ≤ 10 mg/L-regardless of ALB level-received 0 points; those with CRP > 10 mg/L and ALB ≥ 35 g/L were assigned 1 point; and those with CRP > 10 mg/L plus ALB < 35 g/L received 2 points. Patients were assigned to low-risk (0 points), intermediate-risk (1 points), and high-risk (2 points) categories (13, 14).

### 2.4 Outcomes

The primary endpoints of this study were 30-day unplanned readmission and all-cause mortality within one year. The 30-day unplanned readmission was defined as any unscheduled rehospitalization for the initial or related disease within 30 days following discharge, explicitly excluding planned readmissions for chemotherapy, radiotherapy, or surgical procedures (7). The 1-year mortality was defined as death occurring within one year following hospitalization related to LC, regardless of cause.

### 2.5 Ethics

Ethical approval was provided by the institutional review board (approval number: 2024-KY-HY-18). Owing to the retrospective design and anonymized patient data, informed consent requirements were waived by the ethics committee.

### 2.6 Statistical analysis

To evaluate the distribution of continuous variables, the Shapiro-Wilk test was applied. Variables conforming to a normal distribution are presented as mean ± standard deviation (SD). Conversely, variables not following a normal distribution are reported as median with interquartile range (IQR). Continuous variables were compared across groups with one-way ANOVA or the Kruskal-Wallis test, depending on normality. Categorical data are presented as counts (percentages) and were evaluated with the χ² test or Fisher’s exact test, as appropriate.

Kaplan-Meier curves were employed to estimate cumulative event rates of 30-day readmissions and 1-year mortality, with the log-rank test determining significance of differences across mGPS-defined risk groups. Multivariable Cox regression analyses were performed to evaluate the independent association, with results presented as hazard ratios (HRs) and corresponding 95% confidence intervals (95% CIs).

Potential confounding variables were identified through two complementary strategies (1): variables leading to a ≥ 10% variation in regression coefficients upon entry into or removal from regression models, including KPS, NRS 2002, ADL, TNM stage, CA50, WBC count, and hemoglobin; and (2) clinical adjustment for age and metastasis status based on *a priori* knowledge. Multicollinearity among continuous predictors was examined, with variance inflation factors > 10 or tolerance values < 0.1 indicating significant collinearity. Consequently, WBC and hemoglobin variables were excluded from the ultimate model due to collinearity with CRP and ALB, respectively.

Three analytical models were constructed to assess the relationship between mGPS and clinical outcomes: Model 1 was unadjusted; Model 2 was adjusted for age and KPS; and Model 3 was additionally adjusted for NRS 2002, TNM stage, ADL, CA50, and metastatic status. Subgroup analyses assessed the stability of the associations by stratifying patients according to age (< 60 years vs. ≥ 60 years), TNM stage (III vs. IV), metastatic condition (No vs. Yes), NRS 2002 (< 3 vs. ≥ 3), and KPS scores (≥ 70 vs. < 70), using multivariable Cox regression methods. Interaction terms among these subgroups were examined via likelihood ratio tests. The covariates adjusted for in the subgroup analyses were identical to those in Model 3. To handle missing CA50 data, multiple imputation was conducted, generating ten imputed datasets to ensure robust pooled estimates.

All statistical analyses were implemented using R software (version 4.2) and the Free Statistics analytical platform (version 2.2). Statistical significance was defined as p< 0.05.

## 3 Results

### 3.1 Patient characteristics

**Figure 1:**
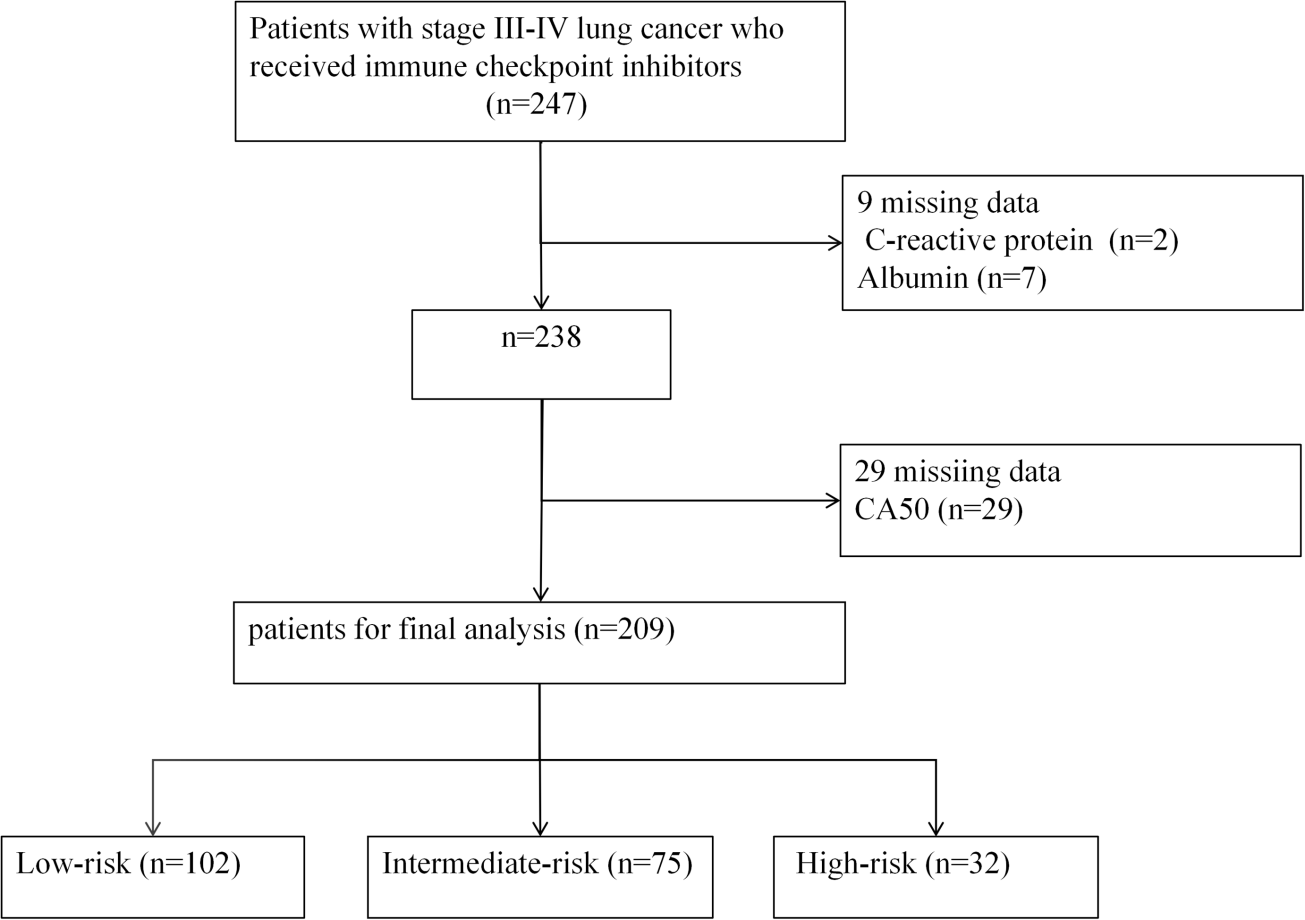
Flowchart of the study cohort.

This study initially enrolled 247 patients with stage III-IV LC receiving ICIs therapy. After excluding patients with missing CRP (n = 2), missing ALB (n = 7), and missing CA50 (n = 29), the final cohort comprised 209 patients (**Figure 1**).

The cohort’s mean age was 64.5 ± 9.5 years; 72.7% were male, and 62.2% were ever-smokers. The 30-day unplanned readmission rate was 35.4% (74/209), and 1-year mortality was 11.0% (23/209) (**Table 1**). The main reasons for 30-day unplanned readmission included infection-related events, pain or fatigue, abdominal pain or diarrhea, and skin rash (**Supplementary Table S1**). By mGPS category, 32 patients were high-risk (2 points) with 24 readmissions, 75 were intermediate-risk (1 points) with 27 readmissions, and 102 were low-risk (0 points) with 23 readmissions. The high-risk group exhibited higher WBC, neutrophil, platelet counts, and CRP levels, and greater malnutrition prevalence. Conversely, red blood cell counts, hemoglobin, ALB, alpha-fetoprotein concentrations, and KPS scores were lower (**Table 1**).

**Table 1:** Baseline characteristics of the study population.

Variables	Total (n = 209)	mGPS			
		Low-risk (n = 102)	Intermediate-risk (n = 75)	High-risk (n = 32)	*p*-value
Age (years), Mean ± SD	64.5 ± 9.5	64.5 ± 8.9	64.2 ± 10.0	65.6 ± 10.1	0.782
Sex, n (%)					0.524
Male	152 (72.7)	72 (70.6)	58 (77.3)	22 (68.8)	
Female	57 (27.3)	30 (29.4)	17 (22.7)	10 (31.2)	
Smoke, n (%)					0.166
No	79 (37.8)	43 (42.2)	22 (29.3)	14 (43.8)	
Yes	130 (62.2)	59 (57.8)	53 (70.7)	18 (56.2)	
Alcohol, n (%)					0.396
No	122 (58.4)	59 (57.8)	41 (54.7)	22 (68.8)	
Yes	87 (41.6)	43 (42.2)	34 (45.3)	10 (31.2)	
TNM stage, n (%)					0.065
III	50 (23.9)	30 (29.4)	17 (22.7)	3 (9.4)	
IV	159 (76.1)	72 (70.6)	58 (77.3)	29 (90.6)	
Diabetes, n (%)					0.932
No	178 (85.2)	87 (85.3)	63 (84)	28 (87.5)	
Yes	31 (14.8)	15 (14.7)	12 (16)	4 (12.5)	
Hypertension, n (%)					0.122
No	149 (71.3)	76 (74.5)	55 (73.3)	18 (56.2)	
Yes	60 (28.7)	26 (25.5)	20 (26.7)	14 (43.8)	
Coronary heart disease, n (%)					0.470
No	197 (94.3)	98 (96.1)	69 (92)	30 (93.8)	
Yes	12 (5.7)	4 (3.9)	6 (8)	2 (6.2)	
Cerebrovascular, n (%)					0.861
No	203 (97.1)	98 (96.1)	73 (97.3)	32 (100)	
Yes	6 (2.9)	4 (3.9)	2 (2.7)	0 (0)	
Distant metastasis, n (%)					0.127
No	43 (20.6)	26 (25.5)	14 (18.7)	3 (9.4)	
Yes	166 (79.4)	76 (74.5)	61 (81.3)	29 (90.6)	
WBC (×10^9^/L), Mean ± SD	6.4 ± 3.5	5.4 ± 1.9	6.5 ± 3.0	9.3 ± 6.2	< 0.001
Lymphocyte (×10^9^/L), Median (IQR)	1.0 (0.6, 1.3)	1.1 (0.7, 1.4)	0.9 (0.6, 1.2)	0.8 (0.5, 1.4)	0.024
Neutrophil (×10^9^/L), Median (IQR)	3.9 (3.0, 5.8)	3.5 (2.5, 4.5)	3.9 (3.0, 6.4)	6.4 (3.8, 9.3)	< 0.001
Red blood cell (×10¹²/L), Mean ± SD	3.8 ± 0.8	4.0 ± 0.7	3.8 ± 0.9	3.5 ± 0.7	0.005
Hemoglobin (g/L), Mean ± SD	116.0 ± 22.6	124.2 ± 19.6	110.4 ± 22.0	103.1 ± 23.9	< 0.001
Platelets (×109^9^/L), Mean ± SD	193.2 ± 101.6	197.2 ± 69.7	173.8 ± 106.0	225.6 ± 156.6	0.045
CRP (mg/L), Median (IQR)	13.0 (3.7, 57.2)	3.6 (1.6, 5.1)	37.2 (23.6, 105.8)	84.0 (43.6, 200.0)	< 0.001
ALB (g/L), Mean ± SD	39.5 ± 6.0	41.5 ± 4.3	40.1 ± 6.5	31.7 ± 2.4	< 0.001
Alpha-fetoprotein (ng/ml), Median (IQR)	3.3 (2.0, 4.8)	3.5 (2.4, 5.5)	3.2 (2.1, 4.6)	2.1 (1.4, 3.0)	0.002
CEA (ng/ml), Median (IQR)	3.7 (1.8, 15.4)	3.1 (1.6, 6.1)	4.9 (2.3, 22.7)	4.4 (1.5, 118.5)	0.036
CA19.9 (ng/ml), Median (IQR)	20.1 (10.1, 34.0)	18.4 (9.9, 28.8)	21.4 (10.4, 44.0)	20.1 (9.3, 48.7)	0.242
CA50 (ng/ml), Median (IQR)	12.9 (7.3, 24.7)	10.8 (7.4, 21.5)	15.3 (7.2, 30.5)	15.7 (7.3, 31.1)	0.331
KPS, n (%)					< 0.001
≥ 70	193 (92.3)	99 (97.1)	72 (96)	22 (68.8)	
< 70	16 (7.7)	3 (2.9)	3 (4)	10 (31.2)	
NRS 2002, n (%)					< 0.001
< 3	178 (85.2)	95 (93.1)	64 (85.3)	19 (59.4)	
≥ 3	31 (14.8)	7 (6.9)	11 (14.7)	13 (40.6)	
ADL, n (%)					0.001
60-100	189 (90.4)	98 (96.1)	69 (92)	22 (68.8)	
40-60	17 (8.1)	3 (2.9)	6 (8)	8 (25)	
< 40	3 (1.4)	1 (1)	0 (0)	2 (6.2)	
30-day readmission, n (%)					< 0.001
No	135 (64.6)	79 (77.5)	48 (64)	8 (25)	
Yes	74 (35.4)	23 (22.5)	27 (36)	24 (75)	
1-year mortality, n (%)					< 0.001
No	186 (89.0)	97 (95.1)	67 (89.3)	22 (68.8)	
Yes	23 (11.0)	5 (4.9)	8 (10.7)	10 (31.2)	

mGPS, modified Glasgow Prognostic Score; WBC, white blood cells; CRP, C-reactive protein; ALB, albumin; CEA, carcino-embryonic antigen; CA19-9, carbohydrate antigen 19-9; CA50, carbohydrate antigen 50; KPS, karnofsky performance scoring; NRS 2002, european nutritional risk score 2002; ADL, activity of daily living scale.

### 3.2 Cumulative incidence of 30-day unplanned readmission and 1-year mortality

**Figure 2:**
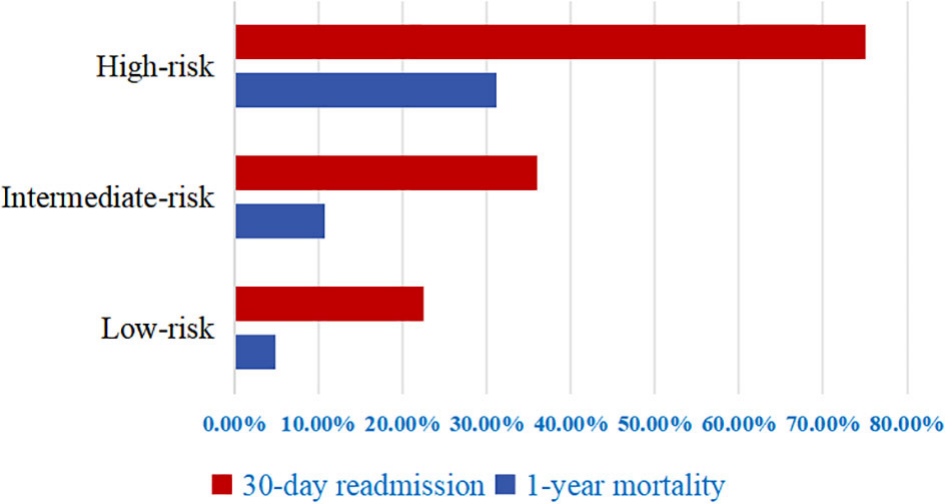
Rates of 30-day unplanned readmission and 1-year mortality by risk groups.

**Figure 3:**
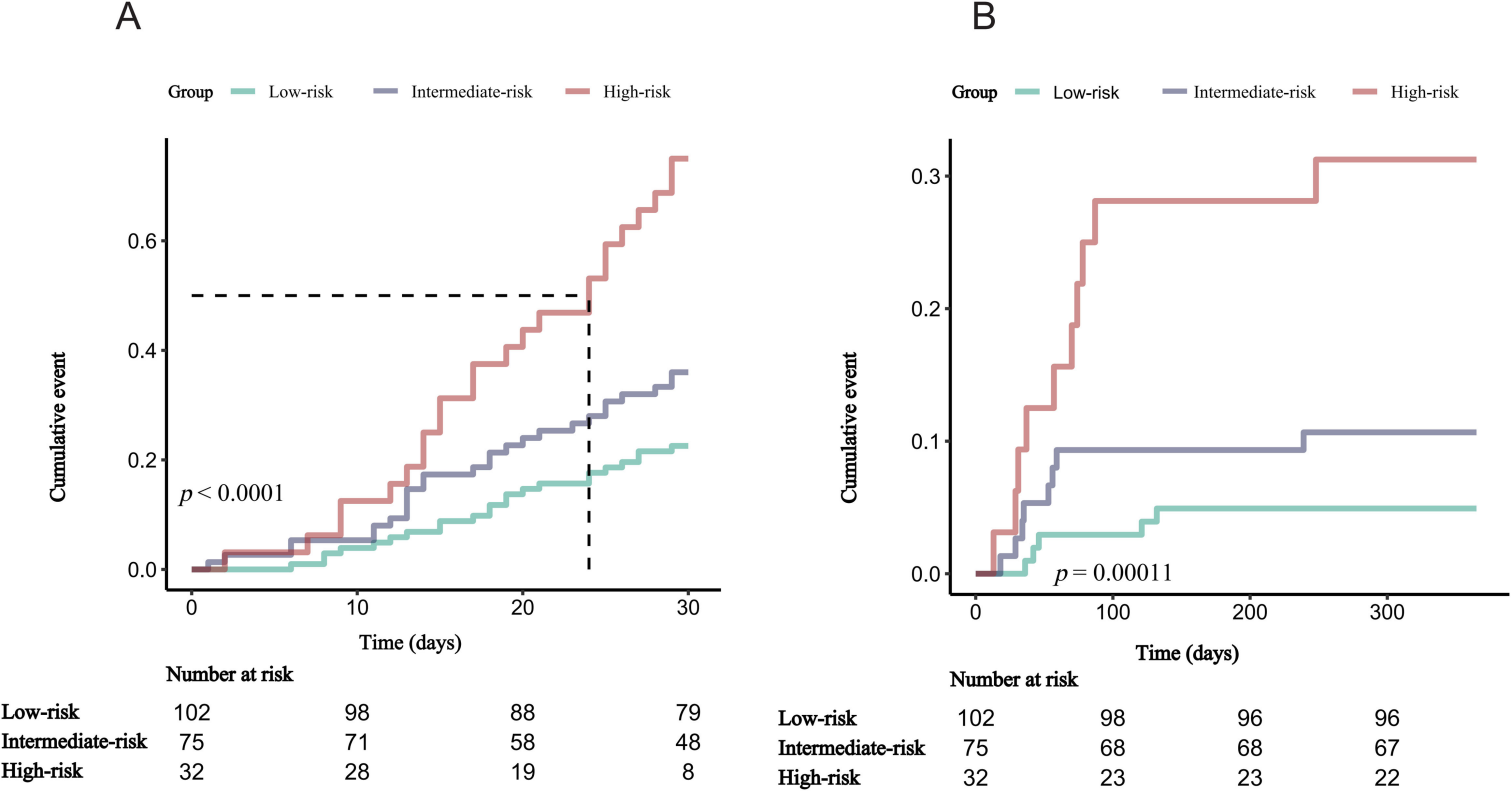
Kaplan-Meier survival curves for 30-day unplanned readmission and 1-year mortality. **(A)** 30-day unplanned readmission. **(B)** 1-year mortality.

**Figure 2** illustrates the distribution of clinical outcomes across risk categories. Patients classified as low-risk exhibited a 22.5% incidence of unplanned readmissions within 30 days, accompanied by a 4.9% rate of mortality within one year. Corresponding outcomes in the intermediate-risk category were higher, at 36.0% and 10.7%, respectively. Patients categorized as high-risk demonstrated the worst prognosis, with rates reaching 75.0% for 30-day readmission and 31.2% for 1-year mortality. Kaplan-Meier survival analyses (**Figure 3**) revealed statistically significant distinctions among the three risk strata for both unplanned readmission within 30 days and mortality at one year (log-rank *p* < 0.001).

### 3.3 Association of mGPS with 30-day unplanned readmission and 1-year mortality

Associations between mGPS levels and clinical outcomes are summarized in **Table 2**. In the unadjusted Model 1, a significant independent positive association was observed between mGPS and unplanned readmission (*HR* 2.18, 95%*CI* 1.62-2.94, *p* < 0.001). For each 1-point increase in mGPS score, the risk of 30-day unplanned readmission increased by 118%. This association remained significant after adjustment for all potential confounders in Model 3 (*HR* 1.72, 95%*CI* 1.25-2.38, *p* = 0.001). Furthermore, when mGPS was modeled as a categorical variable, intermediate-risk patients experienced a 77% increase in the hazard of 30-day unplanned readmission relative to low-risk patients in the unadjusted model (Model 1: *HR* 1.77, 95% *CI* 1.01-3.09, *p* = 0.044), whereas high-risk patients faced a 375% increase (*HR* 4.75, 95% *CI* 2.67-8.44, *p* < 0.001). These associations persisted after full adjustment (Model 3), with high-risk patients still exhibiting a 198% elevation in readmission hazard (*HR* 2.98, 95% *CI* 1.56-5.69, *p* = 0.001).

**Table 2:** Multivariable Cox regression of mGPS with 30-day unplanned readmission and 1-year mortality.

Variable	n.total	n.event_%	Model 1		Model 2		Model 3	
			HR (95%CI)	*p*-value	HR (95%CI)	*p*-value	HR (95%CI)	*p*-value
30-day readmission								
**mGPS as continues**	209	74 (35.4)	2.18 (1.62-2.94)	<0.001	1.93 (1.41-2.63)	<0.001	1.72 (1.25-2.38)	0.001
mGPS								
Low-risk	102	23 (22.5)	1(Ref)		1(Ref)		1(Ref)	
Intermediate-risk	75	27 (36)	1.77 (1.01~3.09)	0.044	1.81 (1.04~3.16)	0.037	1.66 (0.95~2.92)	0.078
High-risk	32	24 (75)	4.75 (2.67~8.44)	<0.001	3.75 (2.02~6.95)	<0.001	2.98 (1.56~5.69)	0.001
*p* for trend				<0.001		<0.001		0.001
1-year mortality								
**mGPS as continues**	209	23 (11)	2.77 (1.61-4.76)	<0.001	2.47 (1.37-4.45)	0.003	2.17 (1.15-4.10)	0.017
mGPS								
Low-risk	102	5 (4.9)	1(Ref)		1(Ref)		1(Ref)	
Intermediate-risk	75	8 (10.7)	2.26 (0.74~6.92)	0.152	2.25 (0.73~6.87)	0.156	1.96 (0.62~6.18)	0.25
High-risk	32	10 (31.2)	7.29 (2.49~21.35)	<0.001	6.03 (1.88~19.26)	0.002	4.66 (1.33~16.35)	0.016
*p* for trend				<0.001		0.003		0.017

Model 1: the unadjusted (crude) model;

Model 2: adjusted for age, karnofsky performance scoring;

Model 3: adjusted as for Model 2, additionally adjusted for NRS 2002, TNM stage, ADL, CA50, distant metastasis.

mGPS, modified Glasgow Prognostic Score; ADL, activity of daily living scale; CA50, carbohydrate antigen 50; HR, hazard ratio; 95%CI, 95% confidence interval; Ref, reference.

Additionally, in the unadjusted Model 1, each 1-point increase in mGPS was associated with a 177% increase in mortality risk (*HR* 2.77, 95%*CI* 1.61-4.76, *p* < 0.001). Although the association was attenuated in Model 3, a 117% increased mortality risk persisted (*HR* 2.17, 95%*CI* 1.15-4.10, *p* = 0.017). Compared with low-risk patients, those in the high-risk stratum had an adjusted 1-year mortality hazard ratio of 4.66 (95% *CI* 1.33-16.35, *p* = 0.016).

### 3.4 Subgroup analysis

**Figure 4:**
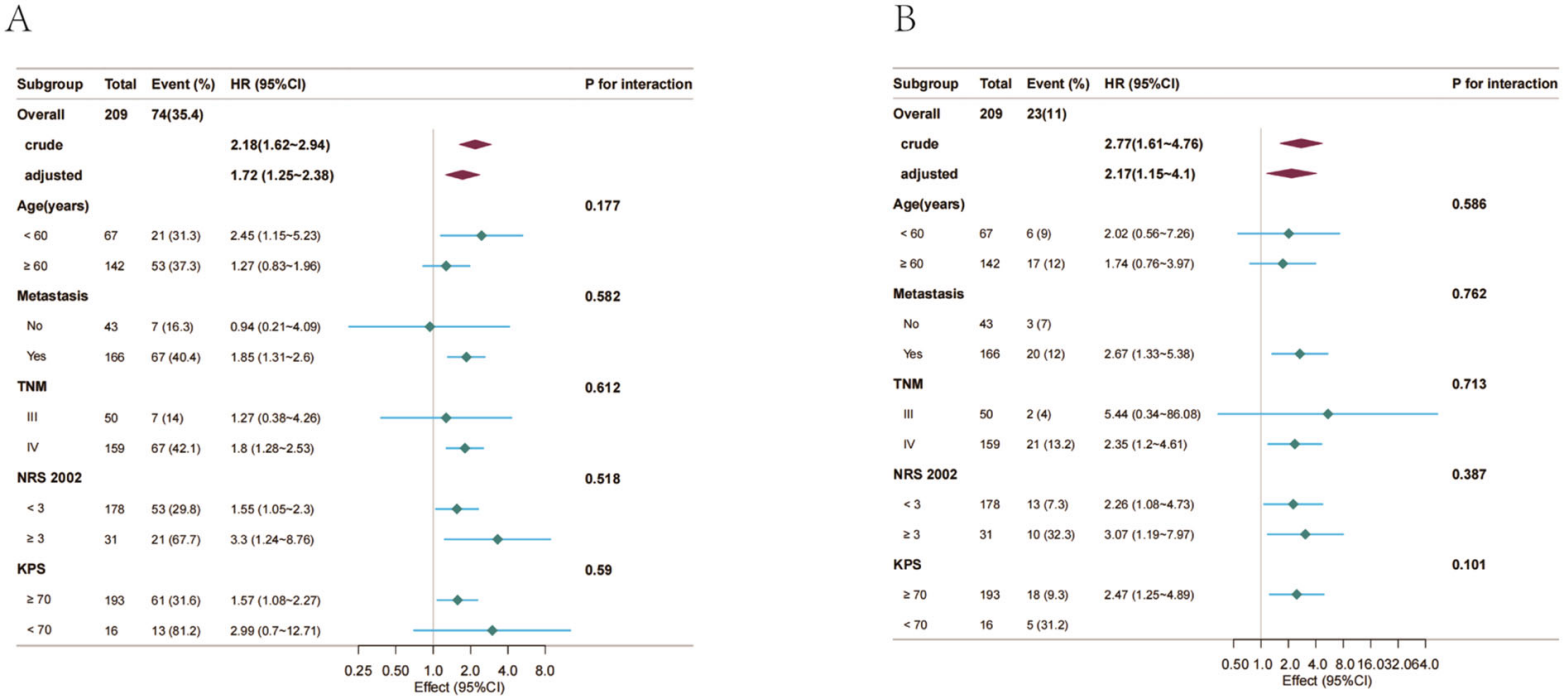
Forest plot of HRs for the 30-day unplanned readmission and 1-year mortality in different subgroups. Adjusted for age, karnofsky performance scoring, NRS 2002, TNM stage, ADL, CA50, distant metastasis. **(A)** 30-day unplanned readmission. **(B)** 1-year mortality. mGPS, modified Glasgow Prognostic Score; LC, lung cancer; ICIs, immune checkpoint inhibitors; KPS, karnofsky performance scoring; NRS 2002, european nutritional risk score 2002; ADL, Activities of Daily Living; CRP, C-reactive protein; CEA, carcinoembryonic antigen; CA 19-9, carbohydrate antigen 19-9; CA50, carbohydrate antigen 50; TNM, tumor-node-metastasis; WBC, white blood cell.

To confirm the stability of mGPS-outcome associations, subgroup analyses with interaction testing were performed. Stratified analyses included age (< 60 years vs. ≥ 60 years), metastatic status (No vs. Yes), TNM stage (III vs. IV), NRS (< 3 vs. ≥ 3), and KPS (≥ 70 vs. < 70), as shown in **Figure 4**. No significant interactions were identified among these predefined subgroups (all *p-*values for interaction > 0.05).

### 3.5 Sensitivity analysis

After multiple imputation of missing data, high-risk patients continued to show substantially increased risks for adverse outcomes. Specifically, in adjusted Model 3, compared with low-risk patients, the high-risk group had a 143% increased risk of 30-day unplanned readmission (*HR* 2.43, 95% *CI* 1.31-4.51, *p* = 0.006) and a 308% increased risk of 1-year mortality (*HR* 4.08, 95% *CI* 1.34-12.43, *p* = 0.025) (**Supplementary Table S2**). Additionally, E-values were 3.64 for 30-day unplanned readmission and 8.79 for 1-year mortality, suggesting that unmeasured confounding was unlikely to alter these findings (**Supplementary Table S3**).

## 4 Discussion

### 4.1 Incidence of 30-day unplanned readmissions and 1-year mortality in LC patients

In this retrospective study of 209 patients with stage III-IV LC receiving ICIs, 35.4% experienced 30-day unplanned readmission, and 11.0% died within one year. These outcomes likely reflect the complex pathophysiology of advanced LC and multifaceted effects of ICIs therapy.

Patients with advanced LC frequently have advanced age, multiple comorbidities, and impaired functional status. Common comorbidities such as pulmonary diseases, cardiovascular diseases, and diabetes further aggravate disease severity (26, 27). Moreover, the immune system’s compensatory capacity is often limited in these patients, and approximately 50% develop immune-related adverse events (irAEs) during ICIs treatment (28). The interactions among tumor biology, immune activation, and ICIs-related toxicities create a complex clinical scenario, significantly influencing treatment outcomes (29).

The 30-day unplanned readmission rate in this study (35.4%) was higher than that reported by Sánchez et al. (23.3%) (7). This discrepancy may be attributed to the higher proportion of patients with stage IV disease (76.1%) in our cohort. Similarly, our readmission rate exceeded that documented by Guven et al. (22.6%) (30). The difference may result from Guven et al.’s inclusion of patients with gastrointestinal and other malignancies. LC carries the second-highest risk for hospitalization and readmission among malignancies (31), possibly explaining the observed discrepancy. Conversely, our readmission rate was lower than that reported by Baş et al. (44.7%) (32). Baş et al.’s higher rate likely reflects their recruitment from a supportive care facility, where patients typically have poorer health status and more comorbidities.

Despite variations in reported 30-day readmission rates, the clinical and economic significance of unplanned readmissions in LC patients is substantial. Each unplanned readmission imposes a direct economic burden of €5,295 per patient (7). Moreover, 30-day readmissions are associated with a 6-fold increased risk of 90-day mortality (33). Thus, early identification of high-risk patients and tailored interventions are crucial.

### 4.2 mGPS as an effective predictor of 30-day unplanned readmission and 1-year mortality

The current analysis indicated that an incremental increase of one point in mGPS independently predicted a 72% increased hazard of unplanned readmission within 30 days, even following adjustments for confounders. Additionally, higher mGPS scores consistently correlated with worse prognoses, including elevated risks of short-term readmission and mortality within one year. Matsubara et al. retrospectively assessed immunonutritional indices, including mGPS, among 24 advanced non-small cell lung cancer patients treated with atezolizumab (24). Their findings similarly established high mGPS as a robust, independent prognostic indicator associated with reduced overall survival, wherein patients identified as high-risk exhibited significantly poorer outcomes relative to low-risk counterparts. Ogura et al. (23) reported that elevated mGPS levels correlated with a 4.17-fold increased hazard for impaired progression-free survival in their univariate analysis; however, their study lacked multivariate adjustments and included only 34 patients, limiting definitive conclusions on confounding influences. Song et al. demonstrated that mGPS is a reliable predictor for overall survival outcomes among non-small cell lung cancer patients (17), further validating the prognostic utility of this biomarker. Despite increasing acknowledgment of systemic inflammation as a critical determinant of LC prognosis under immunotherapy (34, 35), specific investigations regarding mGPS’s predictive value for unplanned readmission remain limited.

Previous studies have indicated that elevated baseline CRP, increased neutrophil-to-lymphocyte ratios, and other inflammatory-nutritional indices, such as Scottish Inflammatory Prognostic Score (SIPS), are associated with unfavorable responses to ICIs therapy and poorer survival outcomes (36–38). In line with the findings of Saal et al., high-risk mGPS assessed at the time of documented disease progression more than doubled the hazard of post-progression death in patients with non-small-cell lung cancer (14). The novelty of this study lies in demonstrating that mGPS is independently associated not only with short-term unplanned readmission but also with increased one-year mortality. Considering that unplanned readmissions impose significant healthcare burdens and negatively impact patient outcomes (39, 40), our identification of a nearly threefold increased hazard of 30-day readmission in patients classified as high-risk underscores the practical value of this readily implementable scoring system for clinical management.

The mGPS is a composite index calculated from CRP and ALB levels. The strong association observed between elevated mGPS levels and poor prognosis in patients receiving ICIs therapy likely stems from the multifaceted biological effects of cancer-related systemic inflammation and malnutrition. Immune-checkpoint inhibitors unleash antitumor immunity, yet the same mechanism can misdirect activated T cells against healthy tissues, producing irAEs. Elevated CRP marks this hyperactive immune state, characterized by amplified T-helper-17 activity and exuberant release of interleukin-6, interleukin-17, and other cytokines that drive the pathogenesis of diverse irAEs (41). Das et al. demonstrated that early clonal expansion of B-lineage cells predicts irAEs risk following combination immune-checkpoint blockade (42). Although their cohort also included patients receiving monotherapy, the finding carries a broader implication: immune-status surveillance can identify individuals at high risk of irAEs. The mGPS, a systemic inflammation index, rises in parallel with analogous immune dysregulation. Severe irAEs—pneumonitis, colitis, hepatitis, or myocarditis—frequently necessitate inpatient intensive immunosuppression and are a direct driver of unplanned readmission (43). Moreover, hypoalbuminaemia may compromise immune regulation and tissue repair, thereby potentiating both the severity and duration of irAEs (44).

Elevated levels of chronic inflammatory cytokines such as interleukin-6 induce neutrophil dysfunction and impair T cell responses (45). Hypoalbuminaemia denotes malnutrition and cachexia, conditions associated with compromised epithelial barrier integrity and diminished production of protective antibodies. This inflammation driven immunosuppression, coupled with malnutrition related barrier defects, markedly increases the risk of opportunistic infections by bacteria, viruses, and fungi in immunocompromised patients with cancer (46). Infection is an important cause of unplanned readmission in patients receiving immune checkpoint inhibitors (47, 48). Consequently, individuals with a high mGPS face an increased likelihood of hospital readmission.

### 4.3 mGPS-guided risk stratification and patient management

Based on peripheral blood-derived parameters, mGPS has been validated as an easily accessible and cost-effective biomarker. Our findings demonstrate that patients with an mGPS score of 2 exhibit a higher risk of adverse clinical outcomes. Consequently, clinicians can utilize this scoring system to identify at-risk patients upon hospital admission (21, 49). Patients with low-risk status (0 points) indicate adequate inflammatory and nutritional profiles, suggesting a low probability of rapid clinical deterioration. Conversely, those with elevated mGPS scores warrant vigilant monitoring for short-term cachexia risk during the initial treatment phase (14). Furthermore, integrating evidence from both the current investigation and prior studies, we contend that patients demonstrating moderate-to-high risk scores warrant intensified clinical surveillance. Serial mGPS assessment at 6 weeks following immune checkpoint inhibitor initiation, when combined with radiographic evaluation, significantly enhances the predictive accuracy for overall survival. This approach enables refined risk stratification of patients with radiographically stable disease into distinct prognostic subgroups, which subsequently informs subsequent therapeutic decision-making (13). Moreover, notably in patients with radiographically confirmed disease progression following ICI therapy yet maintaining low-risk status (0 points), continued treatment with ICI may confer therapeutic benefits. Conversely, individuals with elevated mGPS scores demonstrate marginal clinical benefit regardless of whether they persist with current immunotherapy regimens or transition to alternative therapeutic strategies (14). This observation underscores the critical importance of considering novel mechanism-based interventions—including dual immune checkpoint blockade, antibody-drug conjugates (ADCs), adoptive cell therapy, or comprehensive supportive care—for this high-risk cohort (14).

For patients with elevated mGPS, targeted interventions should focus on mitigating the downstream effects of systemic inflammation and malnutrition. This may encompass the early implementation of nutritional support services, personalized dietary interventions, and anti-catabolic strategies; vigilant monitoring and prompt management of emerging irAEs to prevent clinical deterioration; and heightened awareness of signs indicating infection or cancer-related complications, such as thromboembolic events or effusions. Furthermore, optimizing the management of comorbidities that are exacerbated by inflammation, such as cardiovascular disease and diabetes mellitus, is paramount (50).

## 5 Limitations

This study has some limitations. First, the retrospective observational design and single-center cohort data constrain causal inferences. Secondly, although we performed multivariable adjustments, the absence of data concerning specific types, grades, dosing regimens, timing of therapy initiation for irAEs—the primary drivers of readmission in this population—as well as programmed death-ligand 1 (PD-L1) expression levels, may introduce a degree of confounding bias. Third, standardized illness-severity measures such as Eastern Cooperative Oncology Group performance status (ECOG) or a comprehensive comorbidity index beyond documented conditions were unavailable. Nevertheless, a persistent association between the high-risk group (2 points) and adverse outcomes was observed even after adjusting for multiple potential confounders, indicating that the mGPS remains a valuable and accessible prognostic indicator in this clinical context. Finally, we observed a 30-day readmission rate of 35.4%, which may be attributable to advanced disease stages and complex clinical presentations in this cohort. This potential selection bias limits the generalizability of our findings to all lung cancer patients receiving ICIs, necessitating cautious interpretation of these results. Future prospective investigations with larger sample sizes should prioritize obtaining longitudinal measurements of mGPS dynamics, detailed irAEs profiles, comprehensive patient performance status, and comorbidity data to further elucidate these relationships. Such endeavors would provide more robust insights for clinical management of this patient population across diverse healthcare settings.

## 6 Conclusion

In conclusion, our findings demonstrated that increased mGPS scores were significantly associated with heightened risks of 30-day unplanned hospital readmission and one-year mortality among stage III-IV LC patients receiving ICIs. Consequently, mGPS represents a feasible, clinically useful prognostic instrument. Enhanced clinical surveillance and proactive intervention strategies are recommended, particularly for patients scoring at the highest risk level. However, due to the retrospective study design and absence of longitudinal mGPS measurements, caution must be exercised when interpreting our results. Prospective validation in larger-scale multicenter cohorts remains essential to substantiate these preliminary observations.

## Data Availability

The raw data supporting the conclusions of this article will be made available by the authors, without undue reservation.
