# Supplementary material for: The modified Glasgow prognostic score serves as a robust predictor of unplanned readmission and 1-year mortality in lung cancer patients receiving immune checkpoint inhibitors
Source: Front Oncol. 2026 Jan 21;15:1698848. doi: 10.3389/fonc.2025.1698848 (PMC12867839; doi:10.3389/fonc.2025.1698848)
Supplement: Supplementary file 1 [file Table1.docx]

**Supplementary table S1 Reasons for unplanned readmission within 30 days.**

| **Variables** | **Total (n = 74)** | **mGPS** | | | ***p*-value** |
| --- | --- | --- | --- | --- | --- |
|  |  | **Low-risk**  **(n = 23)** | **Intermediate-risk**  **(n = 27)** | **High-risk**  **(n = 24)** |  |
| Cause of readmission n (%) |  |  |  |  | 0.916 |
| Dyspnea or productive cough | 9 (12.2) | 1 (4.3) | 3 (11.1) | 5 (20.8) |  |
| Nausea and vomiting | 5 ( 6.8) | 1 (4.3) | 2 (7.4) | 2 (8.3) |  |
| Infection-related | 23 (31.1) | 7 (30.4) | 9 (33.3) | 7 (29.2) |  |
| Abdominal pain or diarrhea | 10 (13.5) | 5 (21.7) | 3 (11.1) | 2 (8.3) |  |
| Thrombocytopenia | 4 ( 5.4) | 2 (8.7) | 1 (3.7) | 1 (4.2) |  |
| Skin rash | 10 (13.5) | 4 (17.4) | 3 (11.1) | 3 (12.5) |  |
| Pain or fatigue | 13 (17.6) | 3 (13) | 6 (22.2) | 4 (16.7) |  |

Note: Infection-related encompasses fever, pulmonary infection, neutropenia, and other infections.
