# Supplementary material for: The modified Glasgow prognostic score serves as a robust predictor of unplanned readmission and 1-year mortality in lung cancer patients receiving immune checkpoint inhibitors
Source: Front Oncol. 2026 Jan 21;15:1698848. doi: 10.3389/fonc.2025.1698848 (PMC12867839; doi:10.3389/fonc.2025.1698848)
Supplement: Supplementary file 2 [file Table2.docx]

**Supplementary Table S2 Multivariable Cox regression of mGPS with 30-day unplanned readmission and 1-year mortality.**

| **Variable** | **Model 1** | | **Model 2** | | **Model 3** | |
| --- | --- | --- | --- | --- | --- | --- |
|  | **HR (95%CI)** | ***P*-value** | **HR (95%CI)** | ***P*-value** | **HR (95%CI)** | ***P*-value** |
| **30-day readmission** |  |  |  |  |  |  |
| mGPS as continues | 1.98 (1.5~2.62) | <0.001 | 1.79 (1.34~2.38) | <0.001 | 1.59 (1.18~2.14) | 0.003 |
| mGPS |  |  |  |  |  |  |
| Low-risk | 1(Ref) |  | 1(Ref) |  | 1(Ref) |  |
| Intermediate-risk | 1.91 (1.13~3.21) | 0.016 | 1.94 (1.15~3.28) | 0.013 | 1.77 (1.04~3.01) | 0.038 |
| High-risk | 3.93 (2.27~6.82) | <0.001 | 3.16 (1.76~5.69) | <0.001 | 2.43 (1.31~4.51) | 0.006 |
| *p* for trend |  | <0.001 |  | <0.001 |  | 0.004 |
| **1-year mortality** |  |  |  |  |  |  |
| mGPS as continues | 2.76 (1.69~4.52) | <0.001 | 2.46 (1.45~4.18) | 0.001 | 2.03 (1.15~3.56) | 0.025 |
| mGPS |  |  |  |  |  |  |
| Low-risk | 1(Ref) |  | 1(Ref) |  | 1(Ref) |  |
| Intermediate-risk | 2.11 (0.75~5.92) | 0.157 | 2.08 (0.74~5.85) | 0.165 | 1.84 (0.63~5.34) | 0.279 |
| High-risk | 7.11 (2.7~18.71) | <0.001 | 5.89 (2.09~16.63) | 0.001 | 4.08 (1.34~12.43) | 0.025 |
| *p* for trend |  | <0.001 |  | 0.001 |  | 0.024 |

Model 1: the unadjusted (crude) model;

Model 2: adjusted for age, karnofsky performance scoring;

Model 3: adjusted as for Model 2, additionally adjusted for NRS 2002, TNM stage, ADL, CA50, distant metastasis.
