# Supplementary material for: The modified Glasgow prognostic score serves as a robust predictor of unplanned readmission and 1-year mortality in lung cancer patients receiving immune checkpoint inhibitors
Source: Front Oncol. 2026 Jan 21;15:1698848. doi: 10.3389/fonc.2025.1698848 (PMC12867839; doi:10.3389/fonc.2025.1698848)
Supplement: Supplementary file 3 [file Table3.docx]

**Supplementary table S3 E-value for unmeasured confounding.**

| Item | point | lower | upper |
| --- | --- | --- | --- |
| **Unplanned readmission** |  |  |  |
| RR | 2.11 | 1.36 | 3.21 |
| E-values | 3.64 | 2.06 | NA |
| **1-year mortality** |  |  |  |
| RR | 4.66 | 1.33 | 16.35 |
| E-values | 8.79 | 1.99 | NA |
